# Multi‐Ethnic Analysis of Expression Quantitative Trait Loci (eQTL) in Alzheimer’s Disease: A Focus on Health Disparity Populations

**DOI:** 10.1002/alz.093307

**Published:** 2025-01-03

**Authors:** Nicholas R. Wheeler, Makaela Mews, Tianjie Gu, Lissette Gomez, Patrice L. Whitehead, Kara L. Hamilton‐Nelson, Jose Javier Sanchez, Larry D. Adams, Pedro R. Mena, Takiyah D. Starks, Concepcion Silva, Maryenela Illanes‐Manrique, Michael L. Cuccaro, Jeffery M. Vance, Mario Cornejo‐Olivas, Goldie S. Byrd, Briseida E. Feliciano‐Astacio, Jonathan L. Haines, Gary Beecham, Margaret A. Pericak‐Vance, Anthony J. Griswold, William S. Bush

**Affiliations:** ^1^ Department of Population and Quantitative Health Sciences, Case Western Reserve University, Cleveland, OH USA; ^2^ System Biology & Bioinformatics; Department of Nutrition; Case Western Reserve University, Cleveland, OH USA; ^3^ John P. Hussman Institute for Human Genomics, University of Miami Miller School of Medicine, Miami, FL USA; ^4^ John P. Hussman Institute for Human Genomics, University of Miami Miller School of Medicine, Miami, FL, USA, Miami, FL USA; ^5^ John P. Hussman Institute for Human Genomics, Miami, FL USA; ^6^ Maya Angelou Center for Health Equity, Wake Forest University School of Medicine, Winston‐Salem, NC USA; ^7^ Universidad Central del Caribe, Bayamon, PR USA; ^8^ Neurogenetics Research Center, Instituto Nacional de Ciencias Neurológicas, Lima Peru; ^9^ Dr. John T. Macdonald Foundation Department of Human Genetics, University of Miami Miller School of Medicine, Miami, FL USA; ^10^ Universidad Central del Caribe, Bayamón, PR USA; ^11^ Cleveland Institute for Computational Biology, Department of Population and Quantitative Health Sciences, Case Western Reserve University, Cleveland, OH USA; ^12^ John P. Hussman Institute for Human Genomics, Miller School of Medicine, Miami, FL USA

## Abstract

**Background:**

Despite its high heritability, the genetic mechanisms influencing Alzheimer’s Disease (AD), particularly in health disparity populations like African Americans (AA) and Hispanics (HI), are not fully understood. The lack of ancestral diversity in genetic datasets, notably in eQTL studies that associate genetic variation with gene expression, exacerbates these disparities. Our study seeks to address this gap by comparing the AD interactions of racially and ethnically diverse expression Quantitative Trait Loci (eQTL) effects to investigate the genetic influence on AD in underrepresented populations.

**Method:**

We investigated the impact of AD status on multi‐ethnic eQTLs across 3 diverse ancestral cohorts: AA, HI, and Non‐Hispanic White (NHW). Genotype and gene expression data were collected from samples of each genetic ancestry (AA = 224, HI = 293, NHW = 235), all with known AD status, with a focus on generating whole blood RNAseq data. We modified the TensorQTL pipeline to incorporate an interaction term for AD status to identify eQTL effects potentially induced by disease. We examined the top eQTL association per gene to assess the impact of AD status across the three ancestral cohorts.

**Result:**

Our preliminary analysis reveals striking variations in the impact of AD status on gene expression across AA, HI, and NHW cohorts. Several genes in each ancestry show statistically significant (pv<10e‐8) interaction effects (G X AD) (AA = 11, HI = 5, NHW = 9), indicating that genetic variation within those genes influences gene expression in an AD‐status dependent manner. A number of genes displayed interesting interaction patterns, where the most significant eQTL‐AD interaction was identical in two of the three ancestries studied, suggesting a potential ancestrally‐shared connection to AD risk (AA+HI = 4, AA+NHW = 3, HI+NHW = 5, AA+HI+NHW = 0), though notably there were no genes with induced eQTLs across all three ancestries.

**Conclusion:**

Our study highlights the impact the AD disease process has on genetic variants that alter whole‐blood gene expression. We also highlight differences by population, prompting the development of ethnically diverse gene expression panels that pave the way for more accurate genetic studies in health disparity populations. Our findings also underscore the potential for discovering novel genetic mechanisms influencing AD risk, which could lead to more effective and inclusive therapeutic strategies.

